# Finite element analysis of bone remodeling induced by swelling anchors considering heterogeneous properties

**DOI:** 10.1007/s10237-025-02001-1

**Published:** 2025-08-16

**Authors:** Amirreza Sadighi, Mehrangiz Taheri, Nolan Black, Jordan Stolle, Moein Taghvaei, Madeline Boyes, Sorin Siegler, Thomas P. Schaer, Ahmad R. Najafi

**Affiliations:** 1 Department of Mechanical Engineering and Mechanics, Drexel University, Philadelphia, PA 19104 USA; 2 Department of Clinical Studies New Bolton Center, University of Pennsylvania School of Veterinary Medicine, Kennett Square, PA 19348 USA

**Keywords:** Bone remodeling, Heterogeneous properties, Co-polymeric swelling bone anchors, Hygroscopic swelling, Osteointegration

## Abstract

This study explored the biomechanical behavior of co-polymeric swelling bone anchors and their bone remodeling induction using finite element analysis of a model with heterogeneous properties. First, a hygro-elastic finite element framework was developed to capture the swelling of the bone anchors over time by moisture gain, validated against the data from free swelling experiments. Afterward, finite element models were developed using micro-CT data to capture heterogeneous material properties, and finally, bone remodeling induced by the swelling, acting as a mechanical stimulus, was investigated. The study examined three co-polymeric ratios of methyl methacrylate and acrylic acid (MMA/AA)—80/20, 85/15, and 90/10—and assessed the impact of their associated swelling ratios on bone remodeling and fixation strength. Moreover, in parallel with the numerical investigations, an in vivo study using a sheep model was conducted to evaluate the biocompatibility of these anchors and bone remodeling response to the swelling. The numerical findings highlighted the importance of optimizing swelling ratios to enhance mechanical engagement without causing adverse resorption. More specifically, the results demonstrated that bone regeneration in the region of interest is highly sensitive to the swelling ratio. When the swelling is maintained within an optimal range—such as in the 85/15 composition—favorable densification occurs at the bone–implant interface, enhancing osteointegration. In contrast, excessive swelling (e.g., the 80/20 composition) induces localized overload resorption due to elevated stress concentrations at the interface, which may compromise implant success. Additionally, correlations found between the numerical and in vivo study outcomes supported the notion of an optimal swelling threshold and confirmed the predictive capabilities of the developed hygro-elastic finite element framework. To underscore the importance of favorable bone remodeling in the interface, a push-out study was performed to analyze the fixation strength prior and subsequent to bone remodeling. The significant difference in push-out forces before and after remodeling demonstrates that bone densification at the interface can substantially enhance fixation strength.

**Supplementary Information:**

The online version contains supplementary material available at 10.1007/s10237-025-02001-1.

## Introduction

Bone is a hierarchically structured material, whose adaptation is closely influenced by its trabecular architecture. This adaptation can be evaluated through temporal variations in its morphological and mechanical properties (Tovar 2005; Yoo and Jasiuk 2006). The trabecular architecture of bone is largely shaped by the dynamic remodeling process, which is regulated by its mechanical function. Bone remodeling, governed by mechanical forces, is critical for implant stability (Carter 1984). Bone regeneration is a sophisticated physiological process primarily governed by mechanical stimuli at the cellular level, where osteocytes embedded in the bone matrix sense mechanical loading and translate these signals into biochemical responses that regulate osteoblast and osteoclast activity (Alexander et al. 2024; Klein-Nulend et al. 2012). Fluid shear stress induced by mechanical deformation of the bone matrix serves as a critical stimulus, activating signaling pathways involving integrins and calcium channels, consequently influencing localized bone formation or resorption (Bonewald 2011; Klein-Nulend et al. 2012; Schaffler et al. 2014). According to Wolff’s law, bone adapts dynamically to loading conditions, with resorption occurring in low-stress areas and formation in high-stress regions (Maquet et al. 2012; Rouhi 2012; Wolff 1892). Strain at the cellular level serves as a key mechanical signal for this adaptation (Beaupré et al. 1990). Models incorporating strain energy density (SED) as a stimulus, such as those by Huiskes et al. (1987) and McNamara and Prendergast (2007), have been used to evaluate stress shielding and bone resorption in implant systems.

Excessive or insufficient mechanical loading can disrupt osteointegration, leading to resorption and implant loosening. Early studies by Huiskes and Nunamaker (1984) identified the correlation between elevated stresses and bone resorption near implants, while subsequent work by Van Oosterwyck et al. (2002) established thresholds for stress-induced bone loss. Advanced numerical approaches, including linear and quadratic remodeling models, have since been proposed to improve predictions of bone density changes around implants (Crupi et al. 2004; Li et al. 2007; Liao et al. 2017).

Finite element analysis (FEA) has emerged as a powerful tool for studying bone remodeling and implant performance, offering advantages over experimental methods that are time-intensive, costly, and invasive (Müller 2005; Sharma et al. 2009). FEA enables detailed assessment of stress and strain distributions within bone, aiding in implant design optimization (Weinans et al. 1992). Enhancements to traditional SED-based algorithms, such as incorporating connectivity matrices to model osteoconnectivity, have improved the accuracy of bone ingrowth predictions for porous implants (Cheong et al. 2018; San Cheong et al. 2018).

Additionally, for more accurate analysis results, detailed descriptions of bone geometry and material properties are essential in skeleton finite element models. Computed tomography (CT) provides high contrast between bone and surrounding soft tissues, making it well-suited for capturing precise bone geometry (Bougherara et al. 2009; Zhang et al. 2017). Additionally, the CT value, measured in Hounsfield units (HU), exhibits an approximately linear correlation with bone density and strength. As a result, CT data are widely employed in bone finite element modeling due to their ability to provide both geometric accuracy and information on material properties (Au et al. 2010; Daszkiewicz et al. 2017; Mondal et al. 2022; Sansalone et al. 2010; Stolle et al. 2024).

One area in which bone remodeling analysis is of crucial importance is orthopedic research. Suture anchors and screws are essential in orthopedic procedures for securely reattaching soft tissues to bone (Barber et al. 2003; Cho et al. 2015; Ducic 2001; Frank and Romeo 2018; Harwin 2006) and repairing osteochondral defects or stabilizing fractures (Bazaz and Ferkel 2004; Khazen et al. 2007; Ravenscroft et al. 2013; Taljanovic et al. 2003). However, in low-density bone, such as osteoporotic tissue, insufficient shear resistance can lead to anchor pullout, causing damage to surrounding bone and complicating repairs (Joffre et al. 2017; Nowak 2019; Tjellström et al. 1983; Tumedei et al. 2020; Torstrick et al. 2018). Material properties also play a critical role; titanium anchors, with their high modulus of elasticity, can induce stress shielding and loosen over time (Apostu et al. 2018; Niinomi et al. 2011). Alternatives like PEEK and bioresorbable polymers have been developed to address these issues (Dhawan et al. 2012; Garcia-Gonzalez et al. 2017; Kim et al. 2009; Kurtz and Devine 2007), but similar challenges with pullout resistance remain (Joffre et al. 2017; Tjellström et al. 1983; Torstrick et al. 2018).

Swelling bone anchors, fabricated from co-polymers such as poly(methyl methacrylate-co-acrylic acid) (MMA-AA), have emerged as an innovative solution to address limitations in traditional designs. These anchors, first studied by Greenberg et al. (1978), absorb interstitial fluid and swell in situ, employing co-polymers with hydrophobic and hydrophilic components (Abusafieh et al. 1997b; Siegler et al. 2023). This swelling generates radial stresses, enabling an expansion-fit mechanism for fixation, enhancing frictional resistance against pullout forces (Abusafieh et al. 1997a; Kalidindi and Ahmad 1997; Nien et al. 2001; Sadighi et al. 2023; Taghvaei et al. 2024; Vemuganti et al. 1997). Their unique locking mechanisms further improve fixation strength (Sadighi et al. 2023). These anchors also show promising biocompatibility and osteointegration. Studies by Gualtieri et al. (2000) demonstrated increased bone density around swelling anchors, driven by radial stresses that stimulate bone remodeling (McDonald 2021). Unlike conventional threaded anchors, they can re-establish fixation even after significant dislodgement (Taghvaei et al. 2024), while causing minimal damage to surrounding bone during extraction, making them a superior alternative to traditional designs.

These swelling bone anchors harness hygroscopic swelling to generate controlled radial stresses upon fluid absorption, creating an expansion-fit mechanism at the bone–implant interface (Abusafieh et al. 1997b; Gualtieri et al. 2000; Sadighi et al. 2023; Siegler et al. 2023). These radial stresses, transmitted through the trabecular bone structure, induce localized strains that significantly influence osteocyte mechanotransduction, thereby driving bone remodeling processes. Trabecular bone’s heterogeneous microarchitecture, characterized by variations in porosity, connectivity, and anisotropic mechanical properties, further modulates these mechanical stimuli, critically impacting the distribution and magnitude of stresses that govern cellular responses (Levadnyi et al. 2017; Van Tol et al. 2020). Understanding the interaction between swelling-induced mechanical forces and the trabecular bone architecture is thus essential for optimizing anchor designs and promoting favorable osteointegration outcomes.

To the best of the authors’ knowledge, while most bone remodeling studies assume homogeneous properties, only limited research has explicitly accounted for the geometric complexity and heterogeneity of trabecular bone microarchitecture—highlighting the need for more advanced models to evaluate bone remodeling under various loading conditions. Additionally, although it has been hypothesized that the swelling behavior of co-polymeric bone anchors can act as a mechanical stimulus to induce bone regeneration at the implantation site, no theoretical or experimental studies have confirmed this effect.

This study aims to evaluate the bone remodeling response to radial stresses induced by swelling bone anchors and to establish a robust computational framework for predicting these effects. To investigate the impact of swelling on the bone regeneration, a hygro-elastic framework was developed in Abaqus CAE, and validated against the data obtained from the free swelling experiments conducted on co-polymeric bone anchors with different co-polymeric ratios. Alongside that, the data from a time-zero micro-Ct of ovine lumbar vertebrae was segmented and meshed utilizing Materialise Mimics and 3-matic software, and a 3D model was created to account for the complexity and heterogeneity of trabecular region. Finally, to capture bone remodeling in the implantation site, a Python script was created, incorporating an SED-based remodeling theory, and imported in Abaqus. Using these tools, finite element models (FEMs) were created to investigate the biomechanical behavior of swelling bone anchors and how they would impact bone regeneration in the region of interest. Moreover, in parallel with the numerical investigations, an in vivo study using a sheep model was performed to examine the biocompatibility and remodeling response to the swelling bone anchors. The combined numerical and experimental results revealed how swelling-induced mechanical stimuli influence bone regeneration and fixation performance, informing optimal swelling ratio and implant design.

## Materials and methods

### Materials and free swelling experiments

The fabrication process for the swelling co-polymeric material has been comprehensively explained in the previous research (Abusafieh et al. 1997b, a; Gualtieri et al. 2000; Siegler et al. 2023; Sadighi et al. 2023). Accordingly, only a brief summary is provided here. The cross-linked co-polymer was synthesized from two monomers: methyl methacrylate (MMA), identified as hydrophobic, and acrylic acid (AA), identified as hydrophilic. The cross-linking was facilitated by incorporating di(ethylene glycol) dimethacrylate (DEGDMA) at a fixed volume ratio of $5\%$ to bond two linear co-polymer chains (MMA/AA). To have control over swelling rate, three compositions with varying MMA/AA ratios, specifically 80/20, 85/15 and 90/10, were prepared. Further details on the material characterization of these co-polymers can be found in Gualtieri et al. (2001).

The free swelling experiments have been thoroughly described in Siegler et al. (2023); Taghvaei et al. (2024) and are only briefly summarized here. In these experiments, five samples of swelling bone anchors (from each co-polymeric ratio set) were immersed in bovine serum for 28 days at a constant temperature of $40^{\circ }$C, to simulate in vivo conditions (Baiguera et al. 2010; Gualtieri et al. 2001). Given that these swelling bone anchors would ultimately be embedded in bone tissue, the study acknowledged the real-world constraints and sought to assess the swelling properties, as well as to validate the finite element model. The swelling properties from these experimental sets were used as inputs of swelling finite element models (FEMs), and the dimensional changes were utilized to investigate the accuracy and validity of swelling numerical results. Changes in diameter and length were measured using a micrometer with an accuracy of 0.001 mm.

### Swelling methodology

Hygroscopic swelling occurs when a solid absorbs moisture from its environment, leading to expansion. In cases where the structure is constrained, the swelling induces resulting strains and stresses, referred to as hygroscopic strain and stress. The hygroscopic strain can be approximated, assuming a linear relationship with the absorbed moisture content, as follows:

1 $$\begin{aligned} \varvec{\epsilon _{hs}}=\varvec{\beta _{h}}(\alpha _m - \alpha _{m,ref}), \end{aligned}$$

where $\varvec{\beta _{h}}$ represents the hygroscopic swelling coefficient tensor (m$^3$/kg), $\alpha _m$ is the current moisture content, and $\alpha _{m,ref}$ is the reference moisture content (kg/m$^3$) (Comsol 2017). Based on small deformation theory and linear elasticity, the resulting hygroscopic stress in a swellable implant can be computed using the following equation:

2 $$\begin{aligned} \varvec{\sigma } = C\left( \frac{\nabla \varvec{u}^T + \nabla \varvec{u}}{2} - \varvec{\epsilon _{hs}} \right) , \end{aligned}$$

where $\varvec{\sigma }$ is the stress tensor, $\varvec{C}$ is the elasticity tensor, and $\varvec{u}$ is the displacement vector.

When the swelling of the bone anchor is constrained, compressive pressure is generated at the implant–bone interface, along with tensile hoop stresses in the surrounding bone. The compressive pressure across the interface creates frictional resistance, enabling fixation through an expansion-fit mechanism. Moreover, according to Wolff’s law (Wolff 1892), the tensile hoop stresses could mitigate stress shielding in the bone, potentially inducing bone densification near the implant through a remodeling process.

### Bone remodeling methodology

The current study adopts the traditional bone remodeling theory proposed by Weinans et al. (1992) and Huiskes et al. (1987), with adjustments as recommended in other works (Lin et al. 2009, 2010). Mathematically, the remodeling process can be considered an optimization problem, where the apparent bone density $\rho$ is the objective function, and the mechanical stimulus *S* drives the system toward the reference value *k*. The governing equation is:

3 $$\begin{aligned} \frac{d\rho }{dt} = {\left\{ \begin{array}{ll} B(S-(1+\delta )k),& \text{if } S>(1+\delta )k\\ 0, & \text{if } (1-\delta )k<S<(1+\delta )k\\ B(S-(1-\delta )k),& \text{if } S<(1-\delta )k \end{array}\right. } \end{aligned}$$

Here, *B* is the remodeling rate constant, *k* is the reference stimulus under normal physiological conditions, and $\delta$ defines the width of the “lazy zone”, where bone does not respond to the mechanical stimulus unless a threshold is exceeded (Carter 1984). The three cases in this equation set correspond to bone apposition, equilibrium, and resorption, respectively. The mechanical stimulus *S* driving the remodeling is defined as strain energy density, which is the ratio of strain energy *U* to the apparent bone density $\rho$ (Bourauel et al. 2000; Mellal et al. 2004):

4 $$\begin{aligned} S = \frac{U}{\rho } \end{aligned}$$

The strain energy density for each element, under the assumption of small deformations, is given by:

5 $$\begin{aligned} U = \frac{1}{2} \varvec{\sigma }_{ij} \varvec{\epsilon }_{ij}, \end{aligned}$$

where $\varvec{\sigma }{ij}$ and $\varvec{\epsilon }{ij}$ are the stress and strain components at a material point, respectively.

The system of equations previously discussed, however, does not account for bone resorption caused by overloading and its associated effects. Building on the foundational theory developed by Weinans et al. (1992) and Huiskes et al. (1987), Li et al. (2007) introduced a modified bone remodeling theory that incorporates predictions for bone resorption under overload conditions. This work was later extended by Liao et al. (2017) to include the concept of the lazy zone. As expressed in Eq.6, the relationship between the rate of density change and the mechanical stimulus includes a quadratic term. This adjustment accounts for bone resorption when the mechanical stimulus exceeds a certain threshold, leading to a negative density rate and simulating the effects of overloading (represented in Fig. 4).

6 $$\begin{aligned} \frac{d\rho }{dt} = {\left\{ \begin{array}{ll} B(S-(1+\delta )k) - D(S-(1+\delta )k)^2,& \text{if } S>(1+\delta )k\\ 0, & \text{if } (1-\delta )k<S<(1+\delta )k\\ B(S-(1-\delta )k),& \text{if } S<(1-\delta )k \end{array}\right. } \end{aligned}$$

In the aforementioned equations, *D* represents the overload-resorption constant (Liao et al. 2017; Zheng et al. 2020). The values for the constant bone remodeling rate *B* and the overload-resorption constant *D* were set to 1.0 $(\text{g/cm}^3)^2(\text{MPa} \times \text{time:unit})^{-1}$ and 60 $(\text{g/cm}^3)^3(\text{MPa}^{-2}) \times (\text{time:unit})^{-1}$, respectively, as reported in prior literature based on studies of human bones (Huiskes et al. 1987; Lin et al. 2010; Liao et al. 2017; Weinans et al. 1992). Additionally, a reference stimulus value *k* of 0.004 (*J*/*g*) was used, in accordance with previous findings (Huiskes et al. 1987; Lin et al. 2009; Weinans et al. 1992), where bone apposition occurs above this level and underload bone resorption occurs below it. The lazy zone around the reference stimulus was defined with a $\delta$ value of 10% (Jafari et al. 2022; Lin et al. 2009; Rouhi 2012).

It is noteworthy that while SED-based bone remodeling models do not explicitly simulate biological processes such as osteoblast and osteoclast activity, they have been verified and shown to accurately capture their aggregate effect, namely changes in bone density in response to mechanical stimuli (i.e., SED in this study) (Van Rietbergen et al. 2003; Lanyon and Rubin 1984).

### In vivo experiments

#### Surgical model

This study received approval from the IACUC^1^. Under general anesthesia, twelve skeletally mature ewes (6 males, 6 females) underwent a left retroperitoneal, transpsoatic approach to the lumbar spine. An 8-mm-diameter and 10-mm-deep defect was drilled into the vertebral bodies of L2–L5, and bone anchors were press-fitted. Each animal received four anchors: two 80/20 swelling anchors for mechanical pullout testing, one non-swelling anchor as a control, and one 80/20 swelling anchor, which were both considered for histology. Implant placement into L2–L5 was randomized. Due to the high consistency in trabecular bone density observed across lumbar levels L2–L5 (mean bone volume ratio (BVR) variation < 1%), data from these vertebrae were pooled for analysis, providing a representative and homogeneous dataset for evaluating implant-induced bone remodeling. Six animals were killed after 12 weeks post-operation and the remaining after 24 weeks. At necropsy, vertebrae L2–L5 were processed for mechanical testing and histology evaluation. Specimens designated for micro-CT and histology were fixed in 10% neutral buffered formalin and then transferred to 70% ethanol. Specimens were trimmed to fit into the $\mu$CT scanner (SCANCO $\mu$CT50, SCANCO Medical AG) and scanned at 70 kV, 114 $\upmu$A, with a 250 ms integration time, producing 24.2 $\upmu$m isotropic voxels. Hydroxyapatite phantoms were used for regular calibration, and segmentation was standardized (Sigma = 0.8, Support = 1.0, HU threshold: 200–1000). These scans allowed for quantification of bone regeneration and assessment of swelling behavior over time.

The lumbar vertebral body was deliberately selected as the implantation site for this study due to its relatively uniform trabecular architecture and higher bone density compared to other skeletal regions, which provides a more mechanically robust and consistent environment for evaluating implant performance in the ovine model (Mitton et al. 1997; Watson and McClelland 2018). This choice was motivated by the goal of ensuring consistency in bone quality across specimens, which was critical for isolating the effects of implant swelling on local bone remodeling. Moreover, although vertebral bodies experience multi-axial loads in vivo, their primary loading mode—especially in the lumbar spine—is compressive, balanced by the tensions from muscular and ligamentous, providing a more standardized evaluation platform and a rational basis for evaluating swelling-induced mechanical stimuli in a simplified environment (Adams and Dolan 1995; Rohlmann et al. 2009a, b; Zander et al. 2015).

#### Bone analysis

Micro-CT DICOM images for subchondral bone morphometry were loaded into Dragonfly software (Object Research Systems (ORS) 2024). For micro-CT analysis, DICOM images were imported into Dragonfly software, where manual segmentation was performed to define subchondral bone morphology. For histological sections, regions of interest (ROIs) were evaluated using multiplanar reconstruction, and bone was segmented from the background using Otsu thresholding. At each disk level, the following morphometric parameters were analyzed: bone volume fraction, trabecular thickness, trabecular separation, cortical thickness, cortical porosity, anisotropy, and average total area (cortical $+$ marrow).

**Fig. 1:**
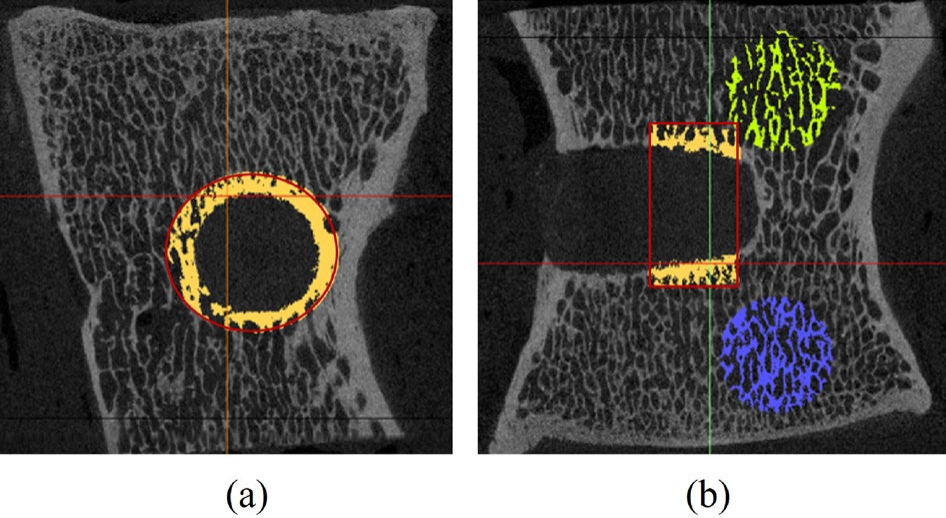
**a** Sagittal view of the hollow cylinder and **b** coronal view of the hollow cylinder (yellow) and spheres (blue and green) in far region. The volume of the hollow cylinder and spheres are $V_0$, and the average bone volumes in them are $V_1$ and $V_2$, respectively. The average bone volume ratios ($V_1$/$V_0$ and $V_2$/$V_0$) will be compared to analyze the impact of swelling on bone remodeling in the bone-implant surfaces.

To quantify new bone formation and assess spatial remodeling, DICOM files were also imported into Dragonfly software. Seven specimens (six at 12 weeks, one at 24 weeks) were included in the analysis. A vertebra with a swelling anchor soaked in fetal bovine serum for 24 h was used as the baseline control. A hollow cylinder (2 mm thick) was defined adjacent to the bone–implant interface to represent the ROI volume ($V_0$). For comparison, two spherical ROIs of equal volume ($V_0$) were placed in trabecular regions far from the implant, as illustrated in Fig. 1. Bone volumes within the cylinder ($V_1$) and the far regions ($V_2$) were computed by applying HU-based thresholding (Aerssens et al. 1998; Gujar and Warhatkar 2020). Bone volume ratios ($V_1/V_0$ and $V_2/V_0$) were compared to assess the local remodeling effects of swelling-induced radial stress. A paired *t*-test was used to evaluate statistical significance. To perform the test, paired BVR values from each vertebra—representing measurements at the interface and distant trabecular sites—were compiled and analyzed in SPSS (IBM Corp. 2025). This pairing allowed for within-sample comparison of local vs. remote bone responses.

#### Histology

Following micro-CT imaging, specimens were plastic-embedded and processed for calcified light microscopy. Sections were stained with Hematoxylin and Eosin (H&E) and Goldner’s Trichrome.

### Finite element models

#### Hygroscopic swelling analysis of bone anchors

**Fig. 2:**
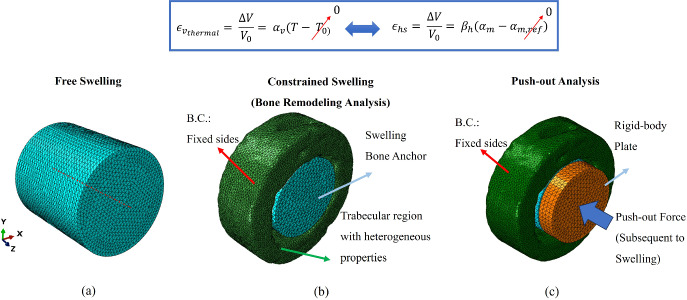
The FEMs of: **a** the free swelling, **b** constrained swelling (bone remodeling analysis) using predefined field on Abaqus FEA software, and **c** push-out analysis.

The hygroscopic swelling simulations were conducted on Abaqus FEA software, using “Predefined Field” to define loading condition, i.e., moisture concentration. This was made possible through the similarity between the thermal expansion relations with those of hygroscopic swelling, as explained in Fig. 2. More specifically, similar to thermal expansion that depends on the thermal coefficient of expansion ($\alpha _V$) and is induced by a change in the current temperature (*T*) from the initial state ($T_0$), the rate of hygroscopic expansion is dominated by the swelling coefficient ($\beta _h$) and determined by the moisture concentration change ($\Delta \alpha = \alpha _m - \alpha _{m,ref}$).

The bone anchors in the model were assigned a fixed diameter and length of 8 mm to match the sizes of the samples used in the free swelling experiments of the bone anchors. In the free swelling models, the boundary condition involved fixing the center axis of the bone anchors. For constrained swelling (bone remodeling analysis), frictional interface with normal behavior was applied, with the coefficient set to be 0.4 aligned with the reports of previous research (Chessin and Driver 1967; Damm et al. 2015; Hughes et al. 2014). Additionally, fixed boundary conditions were applied to the lateral sides of the model. This choice was supported by preliminary finite element analysis of the vertebral model under physiological loading, where an axial compressive load of 500 *N* was applied to the superior surface while fixing the inferior surface, an approach approximating the loading condition aligned with reported loading range in the literature (Smit 2002; Smit et al. 1997; Watson et al. 2014; Windolf et al. 2022). These analyses demonstrated negligible displacement fields at the selected boundary locations, thereby justifying the application of fixed constraints in the remodeling simulations. The swelling behavior of the co-polymeric bone anchors was characterized using experimental data from free swelling tests conducted in bovine serum. These experiments provided measurements of both moisture concentration (weight gain) and volume expansion (radial and longitudinal changes) over the time of swelling. Using this data, the moisture concentration (defined as mass of fluid absorbed per unit volume) and the swelling coefficient (volume change per unit absorbed mass) were calculated for each composition. For the 80/20 composition, the moisture concentration was determined to be 0.1881 kg/m$^{3}$ and the swelling coefficient 1.2287 m$^{3}$/kg. The 90/10 composition exhibited a lower moisture concentration of 0.0487 kg/m$^{3}$ and a swelling coefficient of 0.9032 m$^{3}$/kg. For the 85/15 composition, the values were 0.08672 kg/m$^{3}$ and 0.9920 m$^{3}$/kg, respectively (Sadighi et al. 2025b). These parameters were directly used in the finite element models to simulate the hygroscopic expansion of these anchors and the swelling-induced mechanical response of the surrounding bone.

#### Determining the heterogeneous properties

**Fig. 3:**
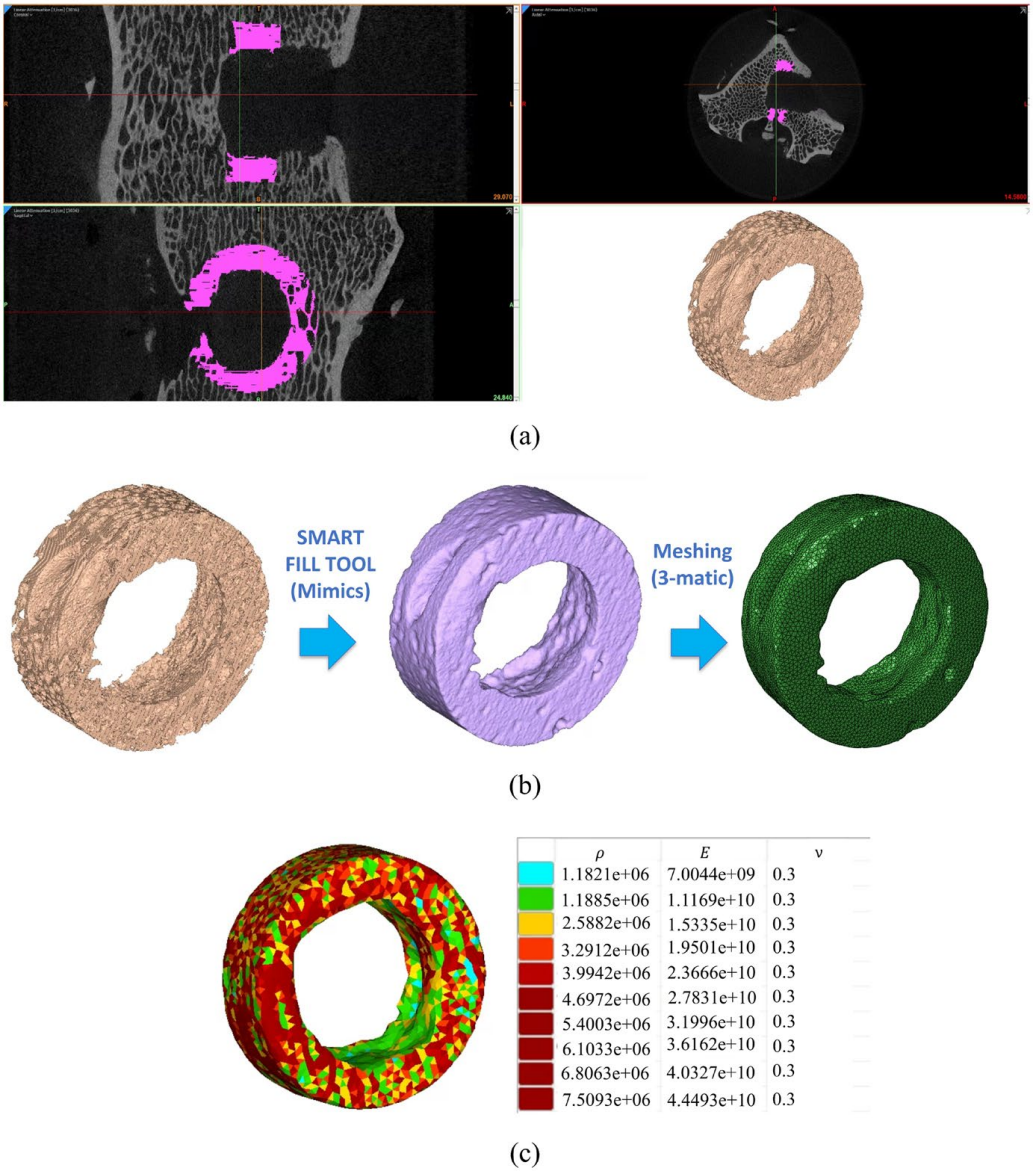
The generation of the trabecular bone FEM with heterogeneous material properties in Materialise Mimics software based on the *HU* in micro-CT: **a** thresholding the micro-CT to create a 3D model, **b** utilizing Smart Fill tool to fill the small gaps between these fine details, and **c** assigning heterogeneous material properties based on HU values.

For computational efficiency, a cylindrical region with a thickness of 5 mm around the hole in the ovine lumbar vertebrae was masked and thresholded using Materialise Mimics software (Materialise 2021). The FEMs focused on a localized region of interest (ROI) surrounding the implant site. This approach was chosen to manage computational demands while capturing the essential local bone response to the hygroscopic expansion of the anchors. As shown in Fig. 3a, the 3D model obtained from the mask contained very fine details, which posed challenges for meshing. To address these issues, the “Smart Fill” function in Materialise Mimics was used to fill the small gaps between these fine details prior to exporting the model to Materialise 3-matic, where the mesh was generated, and the accurate model was obtained through surface reconstruction (Fig. 3b).

To achieve heterogeneous properties, the triangular surface mesh was transformed into a very fine network of tetrahedral volume mesh in Materialise 3-matic to represent the internal material distribution. This yielded approximately 1.3 million quadratic tetrahedral elements, ensuring both numerical accuracy and appropriate mapping of material properties based on Hounsfield units (HUs) derived from the micro-CT scans. In other words, in the actual finite element analysis, the bone mineral density and mechanical properties must be heterogeneous to compensate for the artificially added bone mass density using the Smart Fill feature. Therefore, the model was imported back into Materialise Mimics, where material properties were assigned to each element based on the Hounsfield unit (HU) distribution from micro-CT data. As shown in the HU distribution histogram in Fig. 3c, the internal material of the vertebrae was divided into 10 types, each represented by a different color. The bone mineral density and elastic modulus of the vertebrae were approximately related to the CT value in a linear manner (Ciarelli et al. 1991; Rho et al. 1995; Zhang et al. 2017; Stolle et al. 2024), and were calculated using the following empirical formulas:

7 $$\begin{aligned} \left\{ \begin{aligned}&\rho = 1041395 + 1017 \times \text{HU} \\&\text{E} = -388.8 + 5925 \times \rho \end{aligned} \right. \end{aligned}$$

where *HU* denotes the Hounsfield unit, $\rho$ refers to the bone mineral density (g/m$^3$), and *E* represents the elastic modulus (Pa). Finally, the finite element mesh with heterogeneous material properties was exported as an INP file for use in Abaqus FEA software.

#### Bone remodeling analysis

**Fig. 4:**
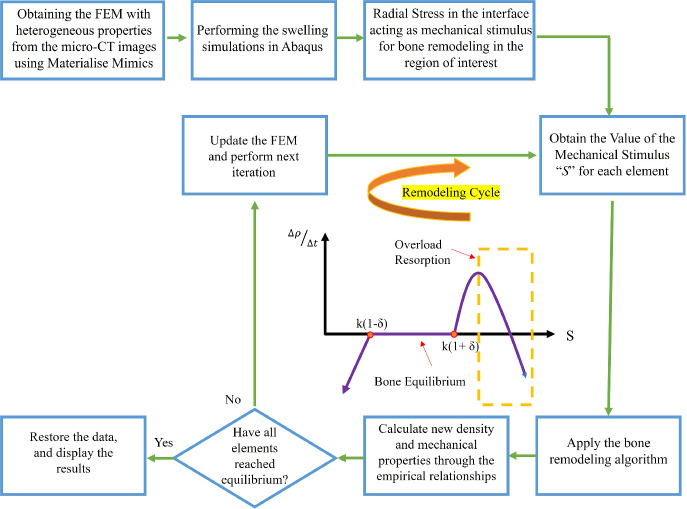
The diagram illustrates the bone remodeling algorithm, which integrates finite element analysis (FEA) for both swelling and bone remodeling processes. Density rate against mechanical stimulus contains the quadratic term to represent overload resorption.

The integration of finite element (FE) analysis with the bone remodeling algorithm offers a computational framework for solving the governing differential equation. In FE analysis, the elements are treated as multiple basic multicellular units (BMUs), each containing mechanosensory cells responsible for detecting strain energy density. The forward Euler time integration method was employed to solve Eq. 3 as follows:

8 $$\begin{aligned} \rho _{m}^{i} = {\left\{ \begin{array}{ll} \rho _{m}^{i-1} + B\Delta t (S_{m}^{i-1}-(1+\delta )k)\\ \quad - D\Delta t (S_{m}^{i-1}-(1+\delta )k)^2 ,& \text{if } S_{m}^{i-1}>(1+\delta )k\\ \rho _{m}^{i-1}, & \text{if } (1-\delta )k<S_{m}^{i-1}<(1+\delta )k\\ \rho _{m}^{i-1} + B\Delta t (S_{m}^{i-1}-(1-\delta )k),& \text{if } S_{m}^{i-1}<(1-\delta )k \end{array}\right. } , \end{aligned}$$

where *i* denotes the time step, and *m* represents the bone element number. The algorithm iterated until the apparent density of each element stabilized, with changes smaller than 2%, signifying the attainment of bone homeostasis. The upper and lower bounds for density were set to 2 and 0.01 g/cm$^3$, respectively, following previous studies (Huiskes et al. 1987; Lin et al. 2009; Weinans et al. 1992). This simulation accounted for both site- and time-dependent processes, including bone resorption and apposition. A time increment ($\Delta t$) of 0.01 s was selected based on prior research to minimize truncation errors (Lin et al. 2009). The computations involved in the remodeling simulation were carried out using a Python script within the Abaqus FEA software (Abaqus 2021). The steps in the bone remodeling study are outlined in the flowchart shown in Fig. 4. As shown, bone remodeling analysis is an iterative process, where each iteration can be considered as the passage of implantation time (Lin et al. 2009, 2010; Zheng et al. 2020). The validity of the framework has already been investigated, and the readers are referred to (Sadighi et al. 2025b) for further details. In-depth discussion regarding the verification of the bone remodeling framework and preliminary results of swelling-induced remodeling using a 2D axisymmetric FEM—whose agreement with in vivo observations provided motivation for this study—are provided in the Supplementary Materials. In the hygro-elastic simulations conducted in this study, to ensure numerical stability of the implicit solver, a small initial increment size (0.001) and automatic time increment size assignment based on force and moment residuals were applied using conservative solver error tolerances. To ensure mesh convergence in this study throughout the verification and primary analyses, seed sizes were iteratively halved and the resulting maximum von Mises stress values were monitored. Refinement continued until stress differences between successive meshes fell below 5%. Due to differences in model dimensionality and complexity, mesh parameters were adapted accordingly, but convergence checks were performed for each FEM model to ensure solution accuracy.

Three different analyses of bone remodeling in the region of interest (ROI) have been conducted for the three different co-polymeric ratios. The reason for doing so is to investigate the impact of swelling ratio on the bone regeneration in the critical segmental defect model of lumbar sheep vertebrae. It has already been reported and shown that the co-polymeric ratio of swelling bone anchors can significantly impact the swelling ratio (Gualtieri et al. 2001), and it has been predicted that in case of excessive swelling ratio, there could be overload resorption in the bone–implant interface, deteriorating the chances of implantation success (Sadighi et al. 2025b).

#### Push-out simulations to investigate fixation strength

To conceptually evaluate the effect of bone remodeling in the region of interest (ROI) on implant fixation, a push-out simulation was performed using a two-step analysis in Abaqus Explicit following the bone remodeling analysis (Fig. 2c). In the first step, the hygroscopic swelling behavior of the bone anchor was simulated to induce radial stresses. In the second step, a rigid-body plate was used to apply an axial displacement-load to the anchor, simulating a push-out scenario. The reaction force was recorded to assess fixation strength, with comparisons made between cases with and without bone remodeling in the ROI. This analysis aimed to illustrate the relative improvement in fixation due to swelling-induced densification, rather than to report absolute fixation strength under physiological conditions.

## Results

### Validation of the hygroscopic swelling analysis using free swelling tests

**Fig. 5:**
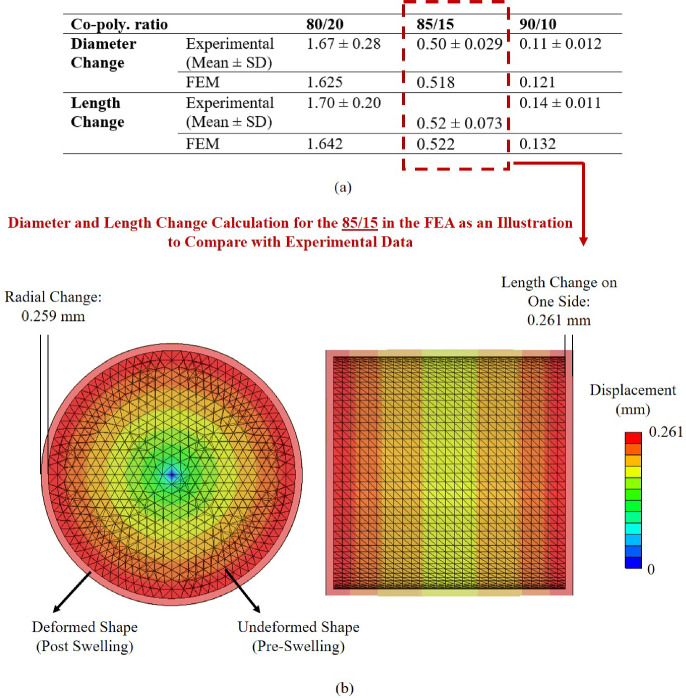
The FEA of free swelling incorporating the swelling properties from experiments. **a** The alignment of the numerical results of dimensional changes with those coming from experiments, proving the validity of the framework and **b** illustration of the approach for calculating the dimensional changes, the diameter and length change of 85/15 composition (As the swelling mechanism is identical across the different co-polymeric ratios, and only the magnitude of diameter and length changes differs, only one composition is presented as a single representative figure).

The validity of the swelling FEA was confirmed by comparison with experimental data. Figure 5 provides the average values and standard deviations for the dimensional changes of samples immersed in bovine serum. The free swelling results obtained from the FEMs align closely with the mean values from the experiments and fall within the standard deviation, thereby validating the proposed computational approach. The approach for calculating the dimensional changes is shown in Fig. 5 for the 85/15 composition as an illustration (aligned with the previous studies (Sadighi et al. 2023, 2025b)).

### Bone remodeling around the swelling bone anchors with different compositions using the FEM with heterogeneous properties

As the results of the previous section suggest the existence of a threshold for swelling ratio beyond which overload resorption may occur, the next step involves applying the hygro-elastic bone remodeling framework to finite element models with heterogeneous material properties. These models incorporate the varying swelling behaviors associated with different co-polymeric compositions.

**Fig. 6:**
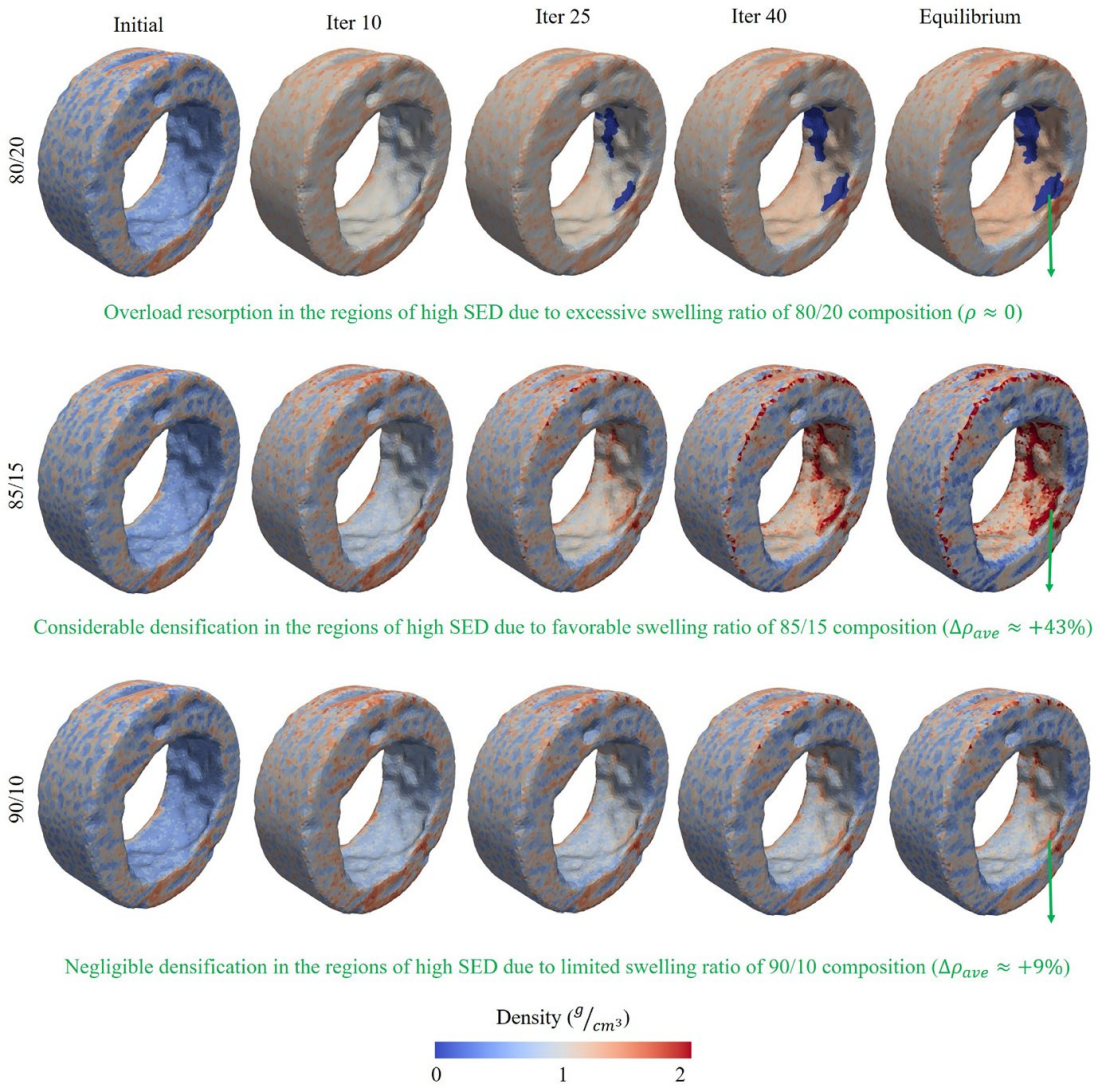
Bone remodeling in the trabecular region of lumbar vertebrae for the three co-polymeric ratios of swelling bone anchors. When the swelling was controlled within a specific limit (for the case of 85/15), favorable densification took place. However, the limited swelling ratio of 90/10 composition did not cause significant densification, and the excessive swelling ratio of 80/20 adversely resulted in overload resorption at the bone–anchor interface.

The bone remodeling induced by the swelling bone anchors is illustrated in Fig. 6. As shown, bone regeneration in the critical segmental defect model of lumbar sheep vertebrae is heavily influenced by the swelling rate and the corresponding co-polymeric ratio of the bone anchors. For example, when comparing the bone regeneration induced by the 90/10 composition with that of 85/15, the constrained swelling ratio in the 90/10 composition resulted in only a modest increase in the density of region of interest. To be more specific, the average density at the interface rose marginally by approximately 9%, from 1.09 g/cm$^3$ to slightly over 1.19 g/cm$^3$. However, the 85/15 composition induced significant densification at the bone–implant interface, with the average density in the interface increasing from 1.09 g/cm$^3$ to over 1.56 g/cm$^3$, representing a 43% increase. To provide further illustration, in the areas with the highest densification rate, the density of some elements increased to over 1.99 g/cm$^3$, approaching the upper limit prescribed in the bone remodeling algorithm.

Nonetheless, in contrast to past expectations and predictions (Abusafieh et al. 1997b; Siegler et al. 2023; Vemuganti et al. 1997), the excessive swelling associated with the 80/20 composition does not seem to be favorable nor effective in improving fixation. As it can be seen, although the swelling has caused an increase in the average density of ROI, the mechanical stimulus associated with this swelling rate would result in adverse overload resorption in some regions at the bone interface, which could greatly risk the chance of implant success. Due to the geometrical complexities of the trabecular region (sharp edges) and lower bone mass densities, the localized stress concentrations due to the high swelling ratio would result in extreme strains at the interface, causing the bone density to drop to the lowest prescribed limit, indicating complete bone resorption. To demonstrate, in regions of high overload resorption (stress concentration), the average density approximately decreased from the initial value of 1.09 g/cm$^3$ to below 0.3 g/cm$^3$.

### Impact of bone remodeling on fixation strength

This section examines how bone remodeling influences the primary objective of the swelling bone anchor, i.e., enhancing fixation to the surrounding bone through mechanically induced integration. As explained in Sect. 2.5.4, two different FEMs of 85/15 composition, one without considering the bone remodeling and the other for the post-remodeling state, were studied to understand the impact of bone regeneration at the interface on the added fixation. It is noteworthy that the main goal is to demonstrate the relative improvement in fixation following favorable bone remodeling.

**Fig. 7:**
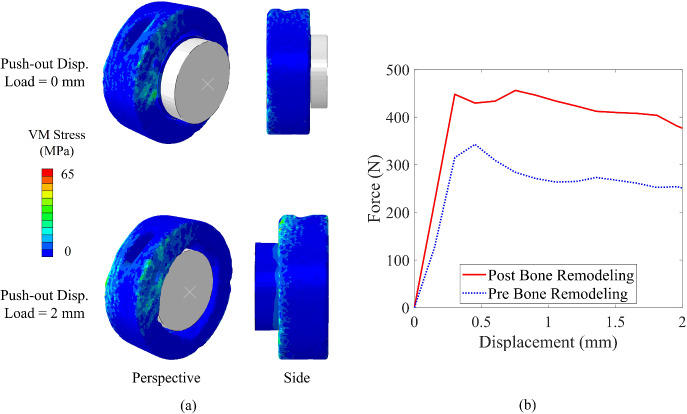
**a** FEA of the push-out of swelling bone anchor, and **b** the force–displacement result of the push-out FEA; Bone remodeling induced by swelling in the surrounding trabecular region would cause a significant rise in the fixation strength.

As it can be seen in Fig. 7, the bone remodeling can cause a considerable difference in the magnitude of fixation strength. Based on the force-displacement result, the maximum push-out force, which is basically the force to overcome the frictional forces existing in the interface with bone and induced by the swelling, was approximately 460 N, whereas this value was around 376 N if no bone remodeling occurred, indicating an approximate 23% increase in the fixation strength. This difference is the direct result of the overall densification in the region of interest, giving rise to the normal (radial) forces on the interface of the co-polymeric anchor over the course of swelling, increasing the value of Coulomb frictional force. This difference could be even greater, as the FEM only considered a 5-mm-thick bone anchor, whereas experimental investigations typically use anchors with a thickness of around 8 mm, which would increase the contact surface and frictional resistance against the push-out (Siegler et al. 2023; Taghvaei et al. 2024).

### In vivo experimental results of bone remodeling around the swelling bone anchors

#### Micro-CT evaluation of bone remodeling

**Fig. 8:**
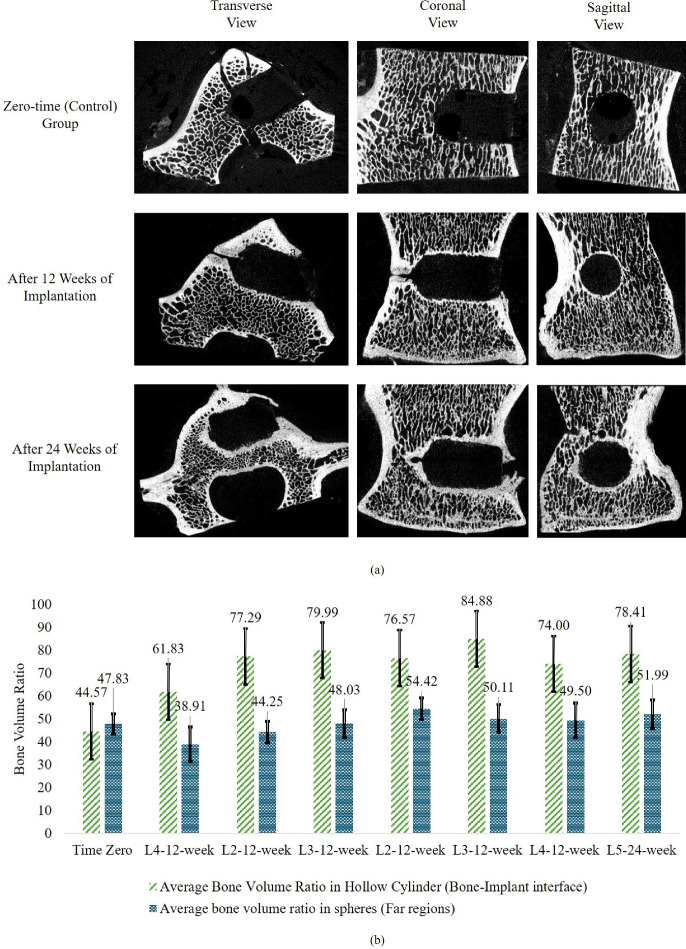
**a** Micro-CT images acquired from the in vivo sheep study at 3 and 6 months post-implantation. Density increases are localized near the bone-implant interface. **b** Average bone volume ratio around the bone-implant interface versus far regions from the interface. There is a significant difference in the average density of bone in the two regions, denoting the densification owing to the radial stresses induced by the swelling.

Figure 8a presents representative micro-CT images acquired from the in vivo sheep study at 12 and 24 weeks post-implantation, alongside baseline (zero-time) images, shown in transverse, coronal, and sagittal planes. As illustrated, the swelling of the anchors led to noticeable bone regeneration and densification around the anchors and in the ROI. This trend, consistent with numerical predictions, indicates that radial stresses resulting from swelling could cause a rise in the average density around the bone–anchor interface. Segmentation analysis further revealed increased bone density in this region compared to intact, distant trabecular areas.

A detailed assessment of the vertebral image analysis following 12- and 24-week implantations using Dragonfly software revealed a significantly greater bone volume ratio surrounding the swelling anchors compared to distant trabecular areas ($P < 0.01$). According to the procedure outlined in Sect. 2.4, the 2-mm-thick hollow cylindrical region at the bone–anchor interface exhibited an elevated bone volume ratio ($V_1/V_0 = 75.759 \pm 7.761\%$), attributed to the anchor’s swelling behavior, relative to the remote intact trabecular region ($V_2/V_0 = 47.537 \pm 5.3531\%$), as shown in Fig. 8b. It was also noted that the zero-time control sample displayed a bone volume ratio near the anchor comparable to that of the distant region. Moreover, the far region after 12 weeks of implantation showed no statistically significant difference in bone volume when compared to the anchor interface of the zero-time control (approximately 46.2%).

#### Histological analysis of the bone–implant interface

**Fig. 9:**
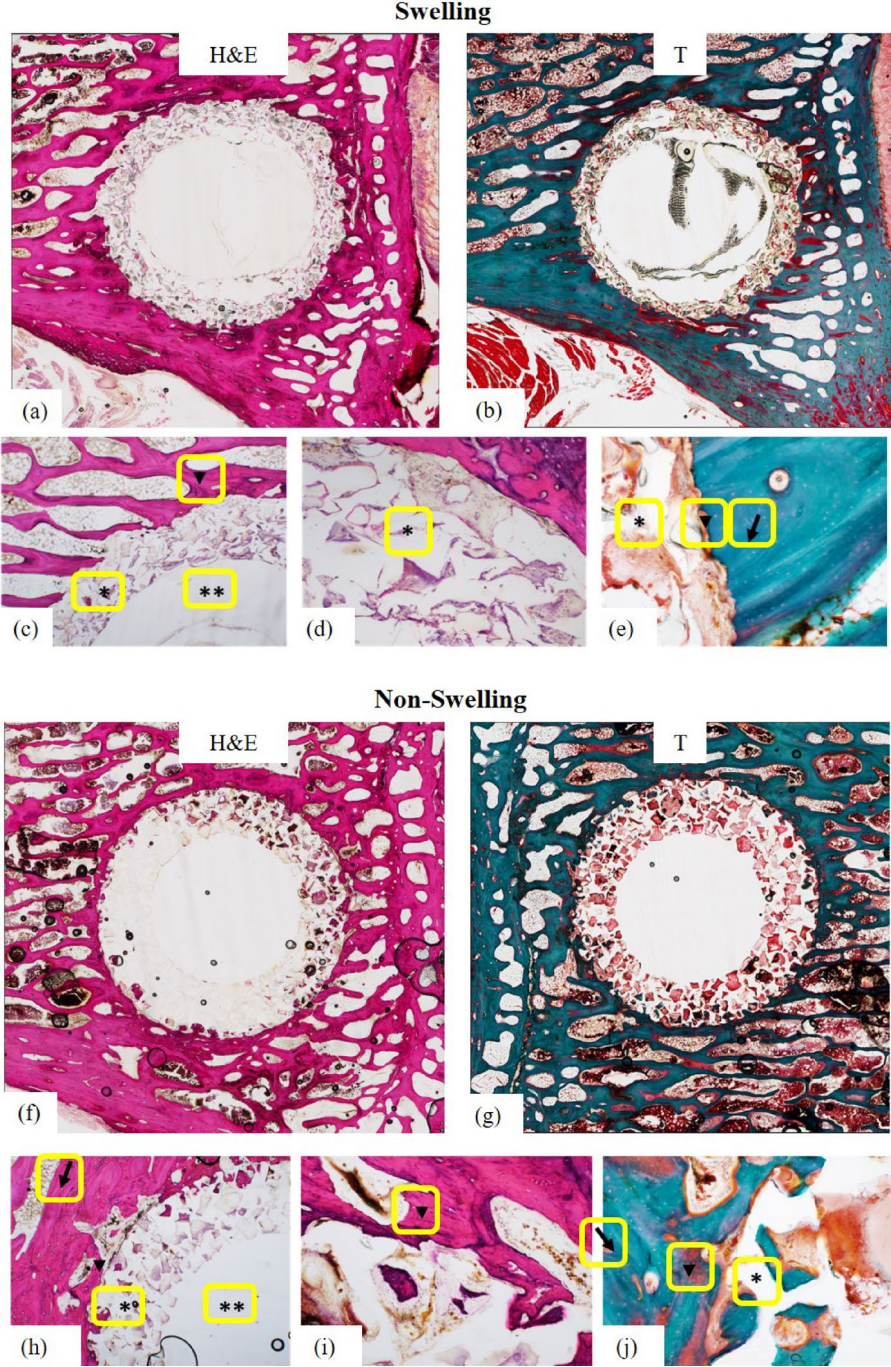
Representative histological sections at 12 weeks post-implantation comparing swelling and non-swelling polymer anchors (Scalebar: 1 mm for the original images; 0.2 mm for the zoomed-in views). (Top) Swelling bone anchor: H&E (**a**, **c**, and **d**) and Trichrome (**b** and **e**) staining show disrupted bone–implant integration, with interrupted apposition, and absence of new bone formation at the interface (arrow). (Bottom) Non-swelling bone anchor: H&E (**f**, **h**, and **i**) and Trichrome (**g** and **j**) staining reveal direct bone–implant contact, elongated osteoid (arrowheads), and substantial new bone ingrowth into the porous structure, confirming improved osteointegration without signs of overload resorption. *porous sleeve, **solid core.

Even though the micro-CT data showed densification in the ROI due to swelling effects, it is crucial to assess bone response and integration at the interface, as this directly influences the ultimate fixation strength. Histological analysis of the bone–implant interface revealed significant differences in bone response between the swelling and non-swelling polymeric anchors. At 12 weeks post-implantation, hematoxylin and eosin (H&E) and Goldner’s Trichrome (T) staining, exhibited in Fig. 9a, b, respectively, revealed limited new bone formation at the interface of bone and the swelling anchors. As evident in Fig. 9c, the porous sleeve (*) and solid core (**) were surrounded by vertebral bone with evident gaps in the pink-stained regions, indicating interrupted apposition,^2^. Within the mature bone adjacent to the bone–implant interface, areas within the endosteal space show evidence of quiescent fibroadipose tissue (*). At higher magnification, Fig. 9d, e provide evidence of adverse bone reaction to the swelling pressure presented by the implant. Highly cellular and poorly organized tissue was observed within the porous matrix (*), consistent with fibrovascular tissue (Fig. 9d) and stained red with trichrome stain (Fig. 9e). In Fig. 9e, the arrowhead indicates the bone–implant interface. Osteoclastic activity is evidenced by the irregular border between the implant and the bone. Unlike the non-swelling implant, here there is no area of new bone formation at the interface between the mature bone (Fig. 9e, arrow) and the porous sleeve (*).

In contrast, at 12 weeks post-implantation, for the non-swelling anchor group, H&E and Trichrome staining showed the porous sleeve (*) and solid core (**) in direct apposition to mature vertebral bone with consistent pink staining, suggesting stable bone integration (Fig. 9h). The presence of mature, elongated osteoid and newly formed bone (arrowheads) at the bone–anchor interface indicated active bone regeneration and integration into the porous scaffold. To further investigate the bone-implant interface, Fig. 9i, j were captured at higher magnification. Adjacent to the bone–implant interface in Fig. 9j, mature bone (arrow) stains blue while immature, poorly ossified/non-ossified, new bone (arrowhead) stains red. Both mature bone and immature bone growth are evident around the bone–anchor interface, indicating bone regeneration at the interface with the non-swelling anchor. The co-polymer matrix is indicated by the *, and new bone growth is pictured within this porous matrix. The newly formed bone appeared less elongated and more mineralized, which in contrast to the histology results of swelling samples. The findings of this section, aligned with numerical results presented in Sect. 3.2, prove that the high swelling rates of 80/20 composition would cause adverse overload resorption at the bone–anchor interface.

## Discussion

This study investigated the biomechanical behavior of co-polymeric swelling bone anchors, providing new insights into their performance, fixation strength and bone remodeling potential. For doing so, a hygro-elastic FE model was created using the predefined fields on Abaqus CAE, to simulate the hygroscopic swelling of the co-polymeric bone anchors. This framework, similar to the previous research, was input the swelling properties extracted from free swelling experiments, and its validity was investigated by comparing the dimensional changes with those of the experimental study (Sadighi et al. 2025b, 2023). Subsequently, a 3D FEM of the bone–anchor interface in lumbar vertebrae in sheep based on the zero-time micro-CT was created on Materialise Mimics software to account for the geometrical complexities and heterogeneity of trabecular bone. Finally, a bone remodeling script, able to capture bone apposition as well as underload and overload resorption, was created to be exploited in the FEA. The primary goal was to evaluate the impact of varying swelling ratios on bone regeneration and how the fixation strength at the bone–implant interface could be impacted by the bone remodeling. Moreover, a concurrent in vivo study using a sheep model was conducted to assess the biocompatibility of these anchors as well as their bone remodeling impact due to the swelling.

The FEM of the trabecular region was developed using Materialise Mimics software, based on the Hounsfield units (HU) of the voxels obtained from micro-CT data. To address meshing challenges and enhance computational efficiency, the Smart Fill tool was employed to close minor gaps between geometrical features. However, this process increased the bone volume in the model. To compensate for the added volume and minimize its influence on the results, heterogeneous material properties were assigned to the FEM. Specifically, the density and elastic modulus of each element were determined according to the HU values of the corresponding voxels, ensuring that the material properties accurately reflected the internal bone structure. Assigning heterogeneous properties based on HU values from micro-CTs improves finite element simulations by accurately capturing bone density variations, enhancing biomechanical predictions. Rho et al. (1995) found that HU values correlate with bone mechanical properties, enabling precise material assignment. Zhang et al. (2017) highlighted that heterogeneous models better replicate stress distribution across cortical and trabecular regions, outperforming homogeneous models. Bougherara et al. (2009) showed that subject-specific models ensures higher fidelity in orthopedic implant simulations by reflecting real-world bone behavior more accurately. This approach has been widely utilized in FEA investigations (Alexander and Weerasooriya 2021; Hammond et al. 2018; Knowles et al. 2022; Stolle et al. 2024), and resolves the limitations of homogeneous models that often oversimplify the complexities of bone behavior (Daszkiewicz et al. 2017).

In Sect. 3.4, the results of in vivo sheep study were explained. Sheep were selected as the in vivo model due to their strong anatomical and physiological similarities to humans. They exhibit comparable metabolic rates (0.22 vs. 0.21 in humans) and possess bone geometry and density characteristics—especially in long bones—that mirror human bone structure and composition (Arens et al. 2007). Mature sheep develop remodeled secondary osteonal bone, resulting in mechanical properties and mineral composition similar to human bone (Nafei et al. 2000). Their bone turnover and remodeling capacities also align closely with human physiology (Martini et al. 2001). For implantation, the lumbar vertebrae provide uniform, weight-bearing sites with consistent bone quality, ideal for evaluating orthopedic biomaterials under axial loading conditions(Smit 2002). These factors make the sheep spine a well-established and translationally relevant site for assessing the remodeling response of swelling bone anchors. Moreover, unlike long bones such as the tibia or femur, which exhibit anatomical variability and seasonally influenced remodeling rates, lumbar vertebrae in sheep offer more consistent bone quality across segments, making them better suited for controlled comparative studies of implant performance (Smit 2002).

Bone’s adaptive response to mechanical stress is central to successful osteointegration (Haga et al. 2009; Huiskes and Nunamaker 1984; Legeros and Craig 1993; Weinans et al. 1992). According to recent research, finite element models incorporating strain energy density (SED) have been widely verified and used to predict localized bone responses, facilitating the design of implants that stimulate favorable remodeling (Lin et al. 2009). Van Tol et al. (2020) demonstrated that mechanical loading and associated strains in the cell level induces fluid flow within bone’s lacunar-canalicular network, which plays a critical role in osteoblast activation and bone formation beyond strain alone. This dual influence of strain and fluid flow supports the notion that swelling-induced radial stress in our study enhanced bone density near the implant. Our findings also align with simulations by Levadnyi et al. (2017), who used FE models to show how mechanical loads around implants influence bone density over time. These insights reinforce that implant design and swelling parameters must be optimized to stimulate bone regeneration and prevent adverse effects like localized stress concentration and resorption.

**Fig. 10:**
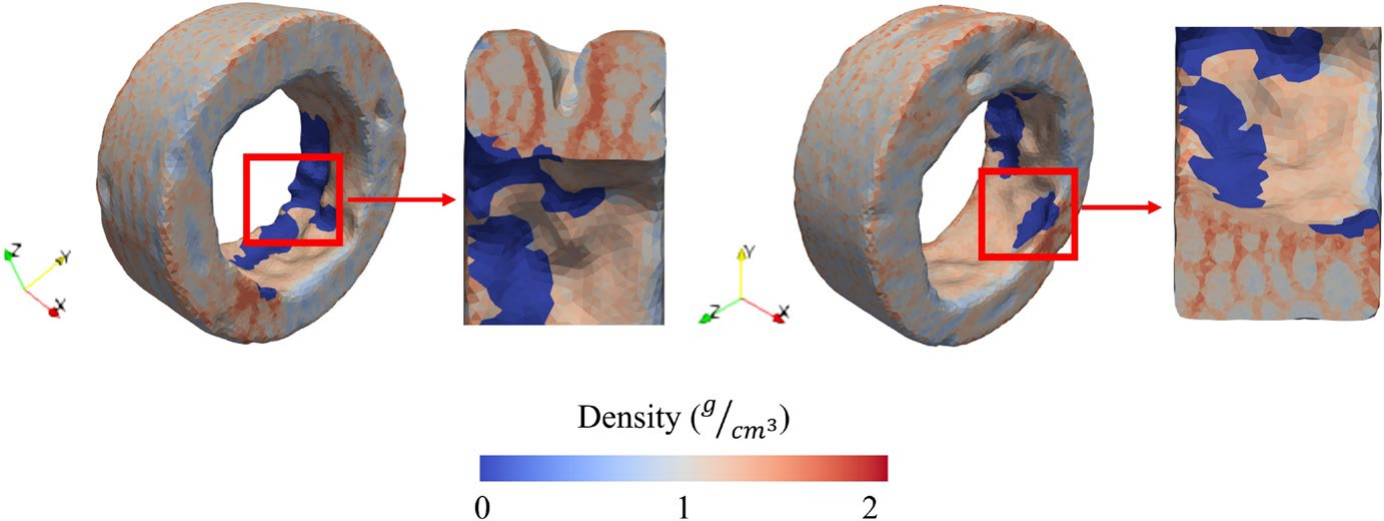
The representation of overload resorption in the bone-implant interface from different viewpoints due to excessive swelling ratio of 80/20 composition.

In Sect. 3.2, the impact of the co-polymeric ratios (material compositions) and their associated swelling ratios on the induced bone remodeling was investigated. Several studies reported that while moderate mechanical stimuli promote bone formation, excessive loading may trigger overload resorption, particularly at implant interfaces (Keaveny et al. 1999; McNamara et al. 2006; Shi et al. 2010; Van Oosterwyck et al. 2002). In this study, the high-swelling 80/20 composition was selected to explore whether elevated radial stresses would enhance fixation or induce a catabolic response. The in vivo findings in this study also revealed localized resorption at the bone–anchor interface under excessive swelling, supporting the hypothesis that mechanical overload can adversely affect bone integrity. Interestingly, through the FEMs considering heterogeneous properties, similar to the previous studies (Sadighi et al. 2025b), and consistent with the in vivo observations, it was also observed that when swelling ratio goes beyond a limit, overload resorption could occur in the bone–implant interface. This would impair the osteointegration as well as adversely impacting the radial stresses induced in the interface, causing a drop in the normal (radial) forces which would provide frictional resistance and fixation strength. For a more in-depth investigation, Fig. 10 has been displayed. As observed, the excessive swelling ratio of the 80/20 composition resulted in overall densification of the surrounding bone (in line with the micro-CT results in Sect. 3.4). However, in certain regions of the bone–implant interface, particularly around sharp edges and deformities where stress concentrations are higher, complete resorption was observed. Although these resorptions occurred within a thin contact area, they could still negatively affect the bone-implant interaction, degrading fixation strength and osteointegration, thereby jeopardizing implant success. In contrast, the densification induced by the 85/15 composition stimulated bone regeneration in some regions, reaching the maximum prescribed density in the algorithm. This would increase the normal force on the lateral surface of the bone anchor, thereby enhancing frictional resistance at the interface and improving fixation strength.

**Fig. 11:**
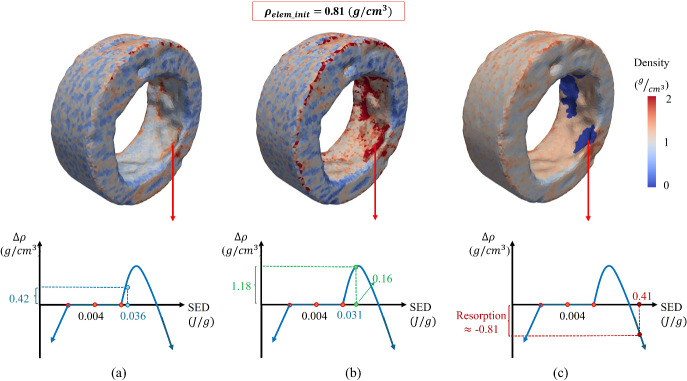
The strain energy density and its associated densification induced by swelling of the bone anchors with **a** 90/10, **b** 85/15, and **c** 80/20 co-polymeric ratios. While the SED caused by 80/20 composition fell in the region of overload resorption, the one for 85/15 would cause favorable bone regeneration, with regions densified to the highest threshold prescribed in the bone remodeling algorithm.

Figure 11 provides a theoretical explanation and the underlying reason for the above-mentioned observations. To understand the phenomenon better, an element-based investigation has been conducted into the elevation in the strain energy density (SED) caused by the swelling, which would induce changes in the density over time ($\textrm{d}\rho /\textrm{d}t$). To do so, an element in a critical region in the interface is chosen, whose initial density is 0.81 g/cm$^3$. As seen in Fig. 11c, the strain that the element underwent due to the swelling of 80/20 composition caused the SED to exceed the overload resorption threshold in a considerable manner, causing a total resorption of the element. For the case of 90/10 composition (Fig. 11a), the density of the same element increases by a maximum of 50%, as the rise in the mechanical stimulus (SED) is not considerable. However, for the case of 85/15, in the areas with the maximum densification, such as the chosen element, the density increases from the initial value of 0.81 to 1.99 g/cm$^3$, indicating an increase by over 145%. This can be seen in Fig. 11b, where the mechanical stimulus is close to the one inducing highest amount of densification over time.

The observations from the in vivo study confirm the projected bone densification due the swelling of the bone anchors. As checked in Fig. 8, the density around the swelling bone anchor compared to the regions far from the interface starts to increase as the implant swells over time and its associated radial stress increases. The same conclusion was made based on the numerical results, where the region of interest became more densified as the simulations got closer to convergence (equilibrium) for all compositions. This densification around the anchor arise from radial stresses associated with the swelling which acted as a mechanical stimulus for bone regeneration according to Wolff’s law and Huiskes’s bone remodeling theory (Huiskes and Nunamaker 1984; Wolff 1892). However, direct comparison of the numerical results with the in vivo data is not possible, as in the simulations, the bone remodeling parameters (i.e., bone density upper and lower limits, bone remodeling constants, reference stimulus and lazy zone region) were based on studies considering human bone, and these parameters are certainly different for that of the sheep.

Histological analysis of the explanted specimens revealed distinct differences in tissue response at the interface between the non-swelling and swelling bone anchors. At 12 weeks, non-swelling implants exhibited consistent bone ingrowth into the porous matrix. Staining showed mature osteoid lining the bone–implant interface and newly formed, immature bone occupying the pores—hallmarks of successful osteointegration without evidence of inflammatory or adverse tissue reactions. Conversely, 80/20 swelling anchors demonstrated limited bone ingrowth and signs of osteoclastic activity along the interface. Histology revealed fibrous tissue infiltration within the porous architecture and disrupted bone contact, indicating an unfavorable remodeling response. While surgical trauma may transiently affect local bone remodeling, no signs of bone resorption were observed at the interface in non-swelling control samples. In contrast, focal resorption was consistently seen around the 80/20 swelling anchors, supporting the role of excessive mechanical stimulus—rather than implantation trauma—as the primary driver of bone loss. These findings suggest that the radial stresses induced by the swelling exceeded the adaptive capacity of the adjacent trabecular bone, resulting in bone resorption at the interface. These observations also align well with numerical results of Sect. 3.2. Even though the 80/20 bone anchor would stimulate remodeling around the bone–anchor interface, at some regions of interface with higher stress concentrations overload resorption could occur due to excessive swelling ratios. It is important to note that micro-CT quantifies bone volume at the millimeter scale within a 2-mm-thick cylindrical region of interest (ROI) using 24.2 ${\upmu }$m isotropic voxels, whereas histological sections provide high-resolution views of the bone–implant interface at the micrometer scale (5 ${\upmu }$m-thick sections; scale bars: 1 mm for overview, 0.2 mm for insets). Despite the difference in spatial resolution and sampling depth, both techniques consistently reveal localized bone densification adjacent to the anchor and focal resorption at the interface in the high-swelling composition. Collectively, the histological and imaging outcomes verify the finite element predictions and reinforce the concept that swelling ratio and its resultant mechanical stimuli must be carefully tuned. The biological response at the bone–implant interface is highly sensitive to the magnitude and distribution of interfacial stresses, which can shift the remodeling balance from deposition to resorption.

Having said all these, while this study demonstrated that there is an optimal range for the swelling rate and the induced radial stresses, defining a fixed optimal swelling ratio remains complex. The mechanical response of the surrounding bone varies by anatomical site, density, and patient-specific factors, all of which influence the remodeling outcome. Nonetheless, based on our numerical and in vivo findings, the compositions close to 85/15 composition appear to lie within a favorable range—promoting bone densification without triggering overload resorption. Compositions near 90/10 may be insufficient to induce remodeling, whereas excessive swelling from 80/20 may risk bone loss. Future experimental and clinical studies could investigate the favorable range for the material composition and also exhibit that the optimal thresholds across different bone types and various physiological conditions.

The post-remodeling analysis of fixation strength underscores the importance of controlled swelling ratio and its consequent favorable bone remodeling. The force–displacement results showed a significant improvement in fixation strength, as the densified bone at the interface provided greater resistance to push-out forces. This relative increase in resistance and fixation strength is attributed to the rise in normal stresses (force) on the lateral surface of the anchors, which enhances frictional resistance compared to the scenario where bone remodeling induced by swelling was not considered. This finding aligns well with the previous findings, where an increase in the density of the artificial bone would cause a rise in the amount of force required to pull out the implants (Taghvaei et al. 2024). This finding is also in agreement with previous studies, where it was reported that the fixation strength of bone anchors is proportionate to the bone density (bone volume fraction) of the surrounding bone (Poukalova et al. 2010; Tingart et al. 2004). It is also noteworthy that based on previous studies, the swelling also promotes bone ingrowth into the porous implant (Sadighi et al. 2025a). The dual effect of bone remodeling in the ROI as well as new bone growing into the implants would significantly increase the bone anchor’s fixation strength.

Anchor pullout is one of the mechanisms of suture anchor failure. It occurs at the anchor–bone interface during arthroscopic rotator cuff repair, resulting in the pullout of the anchors from the bone. Due to the aging of the population, the incidence of rotator cuff tears is growing (Harryman et al. 2003). For rotator cuff repair, arthroscopic suture–anchor repair has gradually replaced open transosseous repair, so suture anchors are now considered increasingly important in rotator cuff tear reconstruction (Barber and Herbert 2013). The majority of patients with rotator cuff tears are over 60 years old, and osteoporosis is very common among them (Tingart et al. 2004). Suture anchors have better pullout characteristics when placed in areas of higher bone mineral density (BMD) (Tingart et al. 2004). However, the use of anchors in patients who are elderly and who may be osteoporotic can potentially increase the likelihood of anchor pullout (Yamada et al. 2007). The bone quality of the greater tuberosity is one of the factors affecting repair integrity (Tingart et al. 2003). In patients with poor bone quality, the failure rate after rotator cuff repair is as high as 68% (Martinel and Bonnevialle 2020). The clinical utility for these novel swelling materials is believed to be most advantageous for applications requiring fixation to cancellous bone, such as soft-tissue reattachment to cancellous bone. More specifically, the swelling bone anchors presented in this study may offer a novel solution by generating radial stresses upon hydration, thereby improving fixation in osteoporotic bone. Moreover, the resulting mechanical stimulus may further promote bone remodeling at the anchor interface (Huang et al. 2024). These features make swelling bone anchors particularly promising for applications such as soft-tissue reattachment in the proximal humerus, where poor bone quality compromises traditional anchor performance.

While this study focused on the short-term effects of swelling-induced radial stresses on bone remodeling, the long-term performance of swelling bone anchors remains an important area for future investigation. The degradation behavior of co-polymeric materials due to swelling over time, and how such degradation affects the mechanical integrity and fixation strength of the anchor, must be evaluated through long-term simulations and in vivo experiments. Moreover, assessing how sustained remodeling patterns evolve beyond initial osteointegration is critical for predicting implant longevity. Future studies should explore the time-dependent mechanical and biological interactions of swelling bone anchors, particularly in weight-bearing anatomical regions, to ensure both structural and design reliability, as well as sustained bone adaptation.

Additionally, while trabecular bone may adapt to increased loading in certain conditions, the results of this study highlight the existence of a potential threshold beyond which mechanical stimulus becomes detrimental. Future studies are needed to experimentally determine this overload threshold in greater detail, and guide the design of swelling anchors with optimal stimulus levels for sustained osteointegration. In addition, while this study utilized established remodeling parameters from widely cited literature (e.g., Weinans et al. 1992), these values were originally derived for specific bones, such as the human femur, and may not universally apply to all anatomical sites or species. Future research should explore the sensitivity of bone remodeling predictions to parameters such as the remodeling rate constant (*B*), overload resorption constant (*D*), reference stimulus (*k*), and lazy zone width ($\delta$). Parametric studies or sensitivity analysis could help recalibrate these parameters for different anatomical contexts and improve the physiological relevance of simulations. Moreover, future computational research could further explore and incorporate biologically driven remodeling mechanisms and the impact of mechanical loading on these mechanisms to more comprehensively capture the multifactorial nature of bone adaptation.

Furthermore, in future studies, integrating artificial intelligence (AI)-driven algorithms could significantly enhance the predictive accuracy of bone remodeling and facilitate optimization of swelling parameters (Bai et al. 2024). For instance, deep neural networks trained on experimental and simulation data may enable rapid prediction of bone density changes and fixation strength across various swelling ratios (Gatineau et al. 2024; Vera et al. 2023). Moreover, reinforcement learning algorithms could be employed to dynamically regulate swelling responses in real time, maximizing osteointegration outcomes under varying physiological conditions and according to the patient-specific bone structure. Incorporating such AI-based strategies would complement the current mechanistic framework and support the design of smarter, more adaptive orthopedic implants.

There are some limitations to this study. Even though many studies utilize FEMs with heterogeneous properties obtained from micro-CT data, one concern lies in the accuracy of HU measurements, which can be affected by beam hardening, scatter, and calibration variability (Han et al. 2022; Li et al. 2024). Additionally, while numerous studies have established strong correlations between HU values, bone mineral density (BMD), and mechanical properties, these relationships can vary based on species, age, sex, anatomical site, and scanning protocols (Duchemin et al. 2008). In this study, to ensure the validity of the HU-based density assignment in the FEM, three 1 cm$^3$ bone cubes were extracted from different vertebral regions, and their experimentally measured densities were found to match the density range derived from HU calibration in the FEM. This agreement supports the validity of the assigned bone properties, despite the known limitations of HU-to-density conversion and the lack of direct pointwise correspondence. However, despite this validity check, and the fact that the current study followed standardized micro-CT procedures, future work should include further experimental validation to strengthen confidence in HU-based material property assignments. Another limitation is that only a narrow region of interest, including trabeculae, was considered. This decision was made to manage computational demands and ensure numerical efficiency. Given that bone remodeling is an iterative process, expanding the finite element model (FEM) beyond the current region would significantly increase the computational cost. The current model already includes approximately 1.3 million quadratic tetrahedral elements, and scaling up the domain would substantially prolong simulation time and may exceed available memory resources. However, boundary effects may have influenced the results, predicting spurious densification, which can be seen in the edges of FEMs in Fig. 6. Therefore, the interpretation of results should focus primarily on the density changes at the bone–anchor interface, since variations near the boundaries may have been artificially induced by stress concentrations resulting from the imposed boundary conditions. Future studies could address this by employing reduced-order models to increase computational efficiency and enable the analysis of larger regions, which would in turn allow to incorporate the effects of more complex physiological loading conditions. Moreover, the interfacial load is expected to vary as the densification in the ROI takes place over time. However, the finite element models (FEMs) assumed a constant pressure resulting from implant swelling. This simplification is justified by the fact that the majority of swelling occurs over a short time, whereas bone remodeling is a long-term process. As a result, the associated radial stresses remain largely unchanged for a considerable period. Nevertheless, a more efficient reduced-order model that couples swelling and bone remodeling could address this limitation. Another limitation lies in the simplification of the swelling model used in the hygro-elastic framework. A linear relationship between moisture concentration and swelling strain was assumed. While the nonlinearity and time dependence of swelling were indirectly captured through the nonlinear moisture uptake profile obtained from free swelling tests, the actual swelling behavior of co-polymeric materials is more complex—characterized by anisotropy and nonlinearity, and influenced by viscoelastic relaxation and fluid diffusion. This assumption was necessary due to the limited experimental data available for calibrating more advanced models. Nevertheless, prior validation of the framework against experimental free swelling and pull-out data supports its accuracy in predicting global interfacial responses (Sadighi et al. 2023). Future work should aim to incorporate nonlinear, anisotropic swelling behavior as more comprehensive material data become available.

## Conclusion

This study investigated the biomechanical behavior and bone remodeling effects induced by co-polymeric swelling bone anchors using a hygro-elastic finite element framework, validated using free swelling experiments. The integration of zero-time micro-CT data allowed for modeling with heterogeneous properties, capturing material-specific responses under varying swelling conditions. Three co-polymeric compositions (80/20, 85/15, and 90/10) were assessed, with a specific focus on the impact of swelling-induced mechanical stimuli on bone remodeling and fixation strength. Moreover, parallel in vivo studies were conducted to investigate biocompatibility and bone regeneration at the interface.

The findings demonstrated that a controlled swelling ratio over time would yield the most favorable results, promoting significant densification at the bone–implant interface. This remodeling would also enhance fixation strength, as evidenced by push-out simulations, underscoring the role of controlled bone remodeling in improving implant stability. Conversely, for the case of excessive swelling, overload resorption was observed in some regions at the bone interface, which could compromise implant success. These findings were further supported by the micro-CT and histological results from the concurrent in vivo study, which showed poor bone integration at the interface with the 80/20 swelling bone anchor, despite overall densification in the ROI due to swelling effects. The study emphasized the need to maintain swelling ratios within optimal limits to prevent stress concentrations and ensure conducive remodeling outcomes.

## Supplementary Information

Below is the link to the electronic supplementary material.

Supplementary file 1 (pdf 347 KB)

## Data Availability

Data sets generated during the current study are available from the corresponding author on reasonable request.

## Footnotes

1 The concurrent in vivo study on sheep spine was conducted with approval from the Institutional Animal Care and Use Committee (IACUC, Protocol $\#$806713) at the University of Pennsylvania adhering to all regulations and institutional policies governing the ethical use of animals in research, and overseen by the Vice Provost for Research.

2 Further detail regarding the hybrid design of swelling anchors, i.e. solid core and porous sleeve, can be found in prior investigations (Siegler et al. 2023; Sadighi et al. 2023).
