# Supplementary material for: Finite element analysis of bone remodeling induced by swelling anchors considering heterogeneous properties
Source: Biomech Model Mechanobiol. 2025 Aug 16;24(6):1937–58. doi: 10.1007/s10237-025-02001-1 (PMC12618450; doi:10.1007/s10237-025-02001-1)
Supplement: Supplementary file 1 — (pdf 347 KB) [file 10237_2025_2001_MOESM1_ESM.pdf]

Finite element analysis of bone remodeling induced by swelling anchors considering heterogeneous properties

Amirreza Sadighi, Mehrangiz Taheri, Nolan Black, Jordan Stolle, Moein Taghvaei, Madeline Boyes, Sorin Siegler, Thomas P. Schaer, Ahmad R. Najafi

Biomechanics and Modeling in Mechanobiology,
Submission ID: efe12bdd-110f-4b8a-ac4d-9b4ccd8f843a

Supplementary Materials

1. Materials and Method: Finite element models

1.1. Model verification for bone remodeling framework

To verify the robustness and accuracy of the developed bone remodeling script, prior to applying to the swelling bone anchor FEMs detailed in section 2.5.1, it was applied to a finite element model of a human femoral mid-shaft section. The objective was to compare the remodeling results at equilibrium (homeostasis) with previously published numerical studies for verification, as well as with clinical and experimental observations for verification.

Figure S1a displays the geometry of the femoral model used in this analysis. An initial uniform bone density of 0.8 g/cm³ was assigned across the domain, consistent with the modeling assumptions adopted by prior researchers. Boundary conditions were defined by fixing the lower surface in the y-direction, with an additional constraint on the lower-left node to restrict motion in both x and y directions. The applied loading conditions, detailed in both Figure S1 and Table S1, replicate in vivo force data previously reported for the human femur during walking and other daily activities. Specifically, Load Case 1 simulates the stance phase of gait, while Load Cases 2 and 3 represent peak loading scenarios during walking.

The finite element simulations were conducted using the Abaqus FEA software, utilizing a “Dynamic-Explicit” solver across three separate analysis steps—each corresponding to a different loading condition—to iteratively calculate the local bone density adaptations. To ensure accurate resolution of density variations and satisfy mesh convergence criteria, a refined mesh comprising approximately 273,000 quadratic triangular elements was adopted. Additionally, in these simulations, due to the presence of very small mesh, mass scaling was used for the purpose of computational efficiency, and the added mass was confirmed to remain below 2% of the total system mass, ensuring computational accuracy.

1.2. Preliminary analysis of swelling-induced radial stress and bone remodeling

Following the validity check of bone remodeling scripts with respect to the previous literature in the human femur FEM, a preliminary FEA regarding the bone remodeling analysis around swelling bone anchors was also conducted to compare the observations with the *in vivo* results before applying the framework to heterogeneous FEMs obtained from micro-CT. To do so, a 2D

axisymmetric model was created, for which the radial load over the time of swelling was imported from earlier numerical investigations conducted in our labs into the hygroscopic swelling of 80/20 swelling bone anchor implanted in 0.8 g/cm³ bone density. This load was imported as "Pressure" load type in the FEM in Abaqus FEA software. Moreover, the sides were fixed, mimicking the boundary condition of the FEM in the prior study. The FEM can be checked in Fig. S1b. Moreover, mesh convergence analysis dictated the utilization of a very fine mesh network with seed size of 0.05 mm (yielding 190k quadratic triangular elements) to ensure the accuracy of the FE solution, as well as the changes in the structure and properties of the bone going through bone remodeling.

Table S1. Applied joint and muscle forces with respective angles under different gait load conditions.

Load Case	Hip Force (N)	Hip Angle (°)	Abductor Force (N)	Abductor Angle (°)	VL Force (N)	VL Angle (°)
Stance Phase (LC1)	2317	24	702.0	28	423.6	0.7
Swing Phase (LC2)	1158	-15	351.0	-8	315.8	0.2
Terminal Stance (LC3)	1548	56	468.5	35	289.6	0.5

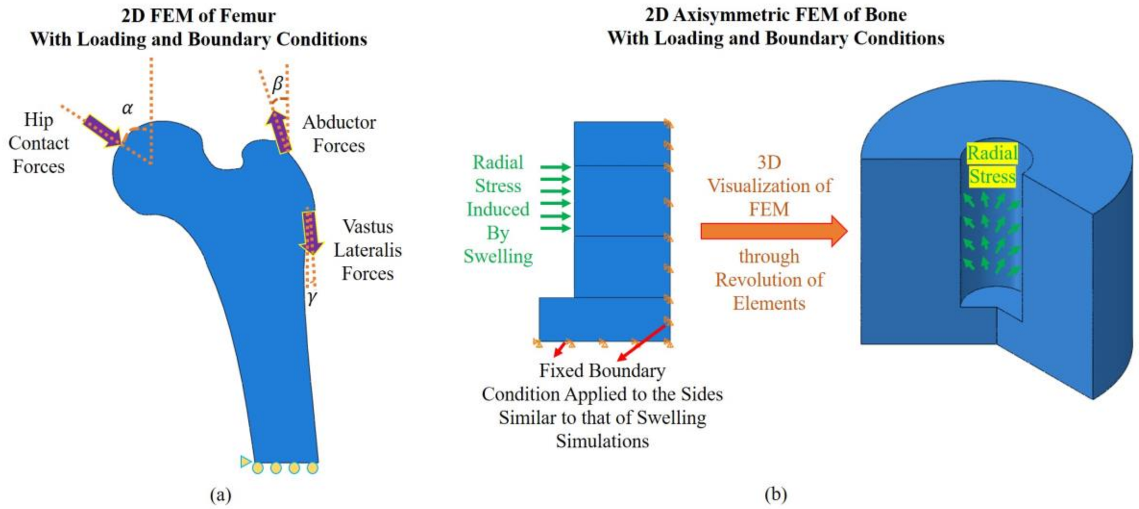

Figure S1. The FEMs of verification studies with loading and boundary conditions: (a) 2D FEM of human femur, and (b) 2D axisymmetric FEM of swelling bone anchors. The analyses are conducted to ensure the reliability of the bone remodeling framework before applying to heterogeneous FEMs obtained from micro-CT.

2. Results

2.1. Verification of the bone remodeling script

As discussed in section 1.1 above, the reliability of the developed bone remodeling framework was initially evaluated through application to a well-established test case, i.e., the human femur. This case has been extensively studied in prior literature, providing both experimental and numerical benchmarks for comparison.

Figure S2a presents the final bone density distribution after the model reached equilibrium (homeostasis). Notably, the resulting density pattern aligns well with clinical CT observations reported in previous studies, including the identification of key anatomical features such as Ward's and Babcock's triangles, consistent with the findings in [1] and [2] (Figs. S2b-d). Additionally, a comparison between our simulation results and those of other established numerical models has been illustrated (Figures S2e-g). Despite differences in geometric modeling and loading configurations, the predicted apparent density distributions showed good agreement. These results demonstrated that the proposed script can reliably capture bone adaptation in response to mechanical loading, validating its use in the subsequent swelling anchor simulations.

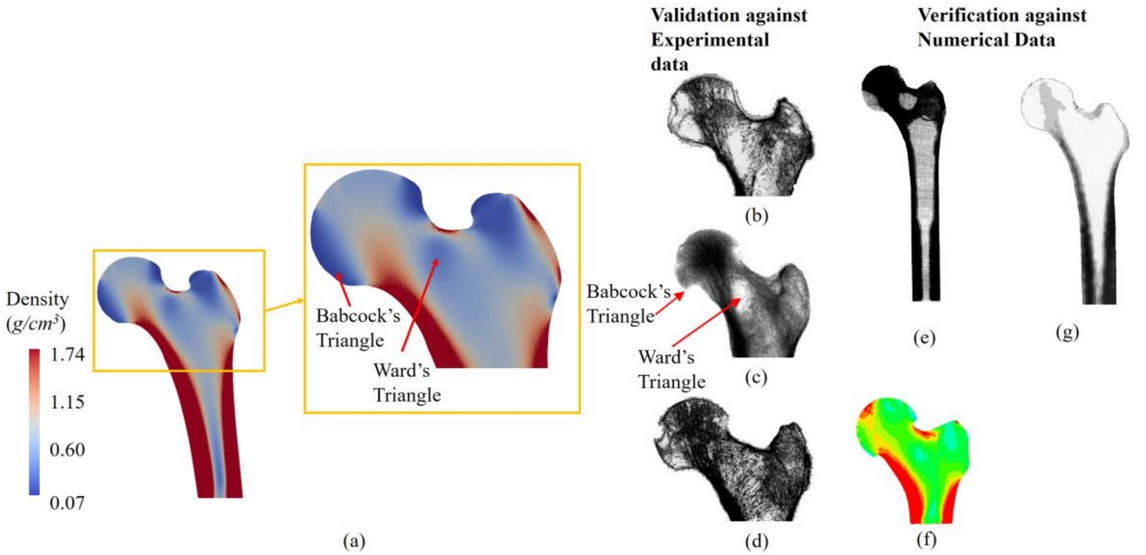

Figure S2. The final structure of human femur after bone remodeling compared with the clinical and numerical data reported in the previous studies; (a) The FE result of the present bone remodeling framework, (b) osteoporotic femur [1], (c) healthy femur [2], and (d) healthy femur [1], (e) Weinans et al. [3], (f) Pérez et al. [4], and (g) Taylor [5]. The final FE result of bone remodeling in this study can sensibly predict the densest and least dense regions of human femur based on the exerted loads as seen in clinical data, and is close to the predictions of bone remodeling in other numerical studies.

2.2. Preliminary assessment of swelling impact on bone remodeling in the bone-anchor interface

As explained in section 1.2 earlier, following the verification of the bone remodeling framework and before investigating the effects of different swelling ratios, a preliminary analysis was performed to evaluate the bone response to the swelling behavior of the 80/20 composition. The objective was to have the results compared with observations obtained from the *in vivo* sheep study.

Figure S3 illustrates the results of the 2D axisymmetric finite element analysis (FEA) of bone remodeling around the 80/20 swelling bone anchor. During the initial iterations (approximately the first 20), localized bone densification was predicted due to the radial stresses exerted on the surrounding bone, as indicated by the darker shades in the model. However, as the remodeling progresses, a narrow region at the bone-anchor interface—where the mechanical stimulus (i.e., radial stress) is the highest—underwent overload resorption. This is evidenced by the reappearance of darker shades concentrated along the interface, which faded into lighter tones moving away from it. Although this resorption is confined to a small area, it may significantly compromise the fixation strength of the anchor, which relies primarily on frictional resistance at the interface.

Notably, this prediction aligns with in vivo micro-CT and histological findings (presented in section 3.4), where, despite bone densification occurring in adjacent areas due to the anchor's swelling behavior, resorption was observed at the interface owing to excessive swelling. Despite the simplifications inherent to the 2D axisymmetric model, and the structural differences between human and ovine bone, this section supports that the remodeling framework can reasonably capture both densification and overload resorption trends. It also motivated further investigation of the impact of swelling ratio on bone regeneration, as detailed in section 3.2.

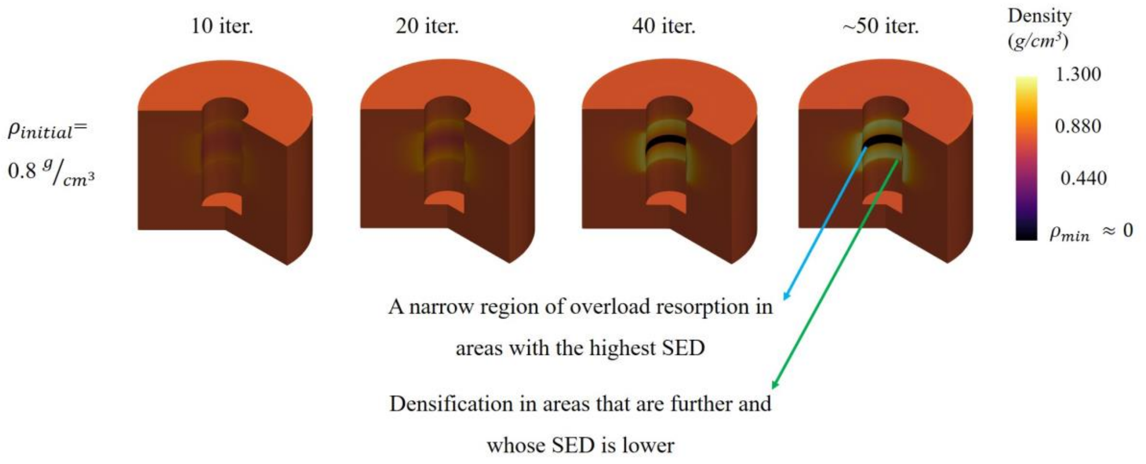

Figure S3. The bone remodeling due to the swelling of the bone anchors with material composition of 80/20. The final converged results suggest that the amount of mechanical stimulus must be over the threshold as overload resorption in a narrow region in the interface is observed compared to areas slightly further.

References

[1] Van Rietbergen, B., Huiskes, R., Eckstein, F. and Rüegsegger, P., 2003. Trabecular bone tissue strains in the healthy and osteoporotic human femur. Journal of bone and mineral research, 18(10), pp.1781-1788.

[2] Jacobs, C.R., Simo, J.C., Beaupre, G.S. and Carter, D.R., 1997. Adaptive bone remodeling incorporating simultaneous density and anisotropy considerations. Journal of biomechanics, 30(6), pp.603-613.

[3] Weinans, H., Huiskes, R. and Grootenboer, H.J., 1992. The behavior of adaptive bone-remodeling simulation models. Journal of biomechanics, 25(12), pp.1425-1441.

[4] Pérez, M.A., Vendittoli, P.A., Lavigne, M. and Nuño, N., 2014. Bone remodeling in the resurfaced femoral head: Effect of cement mantle thickness and interface characteristics. Medical Engineering & Physics, 36(2), pp.185-195.

[5] Taylor, M., 2006, February. Finite element analysis of the resurfaced femoral head. In Proceedings-Institution of Mechanical Engineers Part H Journal of Engineering in Medicine (Vol. 220, No. 2, p. 289). MECHANICAL ENGINEERING PUBLICATIONS LTD.
